# Mixed Methods Examination of Challenging and Bothersome Events in Nursing Virtual Simulations: Comparing Screen-Based and Headset VR Modalities

**DOI:** 10.1177/10468781251401059

**Published:** 2025-11-25

**Authors:** Sayed Azher, Keerat Grewal, Negar Matin, Amanda Cervantes, Caroline Marchionni, Hugo Marchand, Jason M. Harley

**Affiliations:** 1 5620 McGill University, Canada; 2 5620 Research Institute of the McGill University Health Centre, Canada

**Keywords:** virtual simulation, nursing education, emotional arousal, electrodermal activity, technology, usability

## Abstract

**Background:**

Virtual simulations (VSs) are increasingly utilized to train nursing students for clinical practice, yet few tools to evaluate VS quality, and little research has examined specific design elements that impact user experience across different modalities. Comparative evidence between screen-based VS and headset virtual reality (VR) within the same VS scenario is especially limited.

**Purpose:**

The primary goal of this study is to identify challenging/bothersome events in a VS, compare how these events manifest in screen-based versus headset VR, and examine their impact on electrodermal activity (EDA; physiological arousal), performance, usability, and cognitive load.

**Methods:**

A mixed-methods approach was applied to analyze audio-video and EDA data from 20 third-year nursing students during a VS. Objective performance scores were generated by the OMS software, while self-reported data on usability and cognitive load were collected through post-simulation surveys.

**Results:**

Six challenging/bothersome event categories were identified: software-related restrictions and bugs, confusion/lack of success, negative affect, technical errors, neglect of instruction, and other. Results revealed that encountering challenging/bothersome events significantly increased students’ EDA (an indicator of physiological arousal), possibly reflecting frustration/confusion. Furthermore, while no single challenging/bothersome event category predicted performance or usability relative to others, we found that software-related restrictions and bugs were particularly critical, significantly increasing extrinsic cognitive load compared to technical errors.

**Conclusions:**

Our findings highlight specific VS elements that hinder students’ experience, directly pointing to areas for future improvements. We also provide valuable insights for educators and developers to enhance virtual learning environments in nursing education by addressing how to reduce cognitive load and improve performance and usability. Future research should develop VS-specific usability tools and replicate this study in diverse contexts to further refine virtual learning experiences.

## Background

Nursing education necessitates extensive preparation before clinical practice (Das, 2023). This training often occurs through realistic simulations (Mueller et al., 2017; Pickering & Swinnerton, 2019). Simulations can occur, in-person, virtually, or both (Azher et al., 2023). In-person simulations may utilize role-playing actors, manikins, and medical equipment (Azher et al., 2023). However, in-person simulations are resource-intensive and costly (Azher et al., 2023).

Virtual simulations (VSs) are an alternative to in-person simulations and are most cost- and resource-efficient (Azher et al., 2023). As defined in the Healthcare Simulation Dictionary, VSs involve real people operating simulated systems and may include simulations that exercise motor, decision-making, or communication skills (SSH, 2025). VSs can be accessed through two different primary modalities: by using a mouse and keyboard (screen-based VS) or using a virtual reality headset (headset VR) (Azher et al., 2023; Chang & Weiner, 2016; Radianti et al., 2020). Headset VR provides a more immersive virtual environment (Chang & Weiner, 2016; Radianti et al., 2020). For example, students can use their bodies to navigate within the simulation (e.g., real-life movement is mirrored in VR). While screen-based VS is the more popular modality, headset VR use is increasing, in part due to the declining cost of VR hardware compared to earlier years of adoption (Al-Ansi et al., 2023).

### Virtual Simulation in Nursing Education

Virtual simulations are utilized in nursing education, often as an adjunct to traditional training (Aebersold, 2018; Azher et al., 2023). They give students the opportunity to practice certain healthcare scenarios and procedures in their own time and receive automated, and personalized feedback (Azher et al., 2023). General benefits of VSs in nursing education include providing an interactive learning environment rich with personalized feedback (Azher et al., 2023). Such environments and feedback have been met with high student perceptions of self-efficacy and user satisfaction (Medel et al., 2024; Sharoff, 2022). VSs have also been shown to enhance performance (Sim et al., 2022), foster competencies in psychomotor skills (Ahmed et al., 2025; Wilson-Sands et al., 2015), improve general conceptual knowledge (Kleinert et al., 2015; Swan & Giordano, 2023), procedural skills (Aebersold et al., 2018; Yoon et al., 2024), and clinical decision-making (Sim et al., 2022).

### Virtual Simulation Modality Comparison

Previous research comparing screen-based VS and headset VR modalities has shown that they are similar in terms of performance benefits, cognitive load requirements, and usability (Azher et al., 2023). Despite this, limited work has directly compared screen-based VS and headset VR *within the same study* and *using the same VS platform*, allowing for control over scenario content, difficulty, and design features across modalities (Azher et al., 2023; Lavoie et al., 2025). Currently, modality-related differences (e.g., efficacy, usability) are drawn from *separate studies* utilizing either screen-based VS or headset VR with a heterogeneous pool of software and hardware, limiting the validity of conclusions synthesized (Allcoat & Mühlenen, 2018; Yoganathan et al., 2018). Much of the previous work is also quantitative, relying on self-report measures and/or performance metrics and lacks a qualitative exploration of VSs and their features (Azher et al., 2023; Brown et al., 2021; Mallik et al., 2021). For example, recent research evaluating user experience in VSs relies heavily on self-reported measures, drawing from non-VS specific tools such as the System Usability Scale (SUS) (Azher et al., 2023; Brown et al., 2021; Webster & Dues, 2017). To illustrate, the SUS contains general items such as: *I found the various functions in this system were well integrated* (Brooke, 1996), and is thus not tailored to the VS environment, limiting nuanced investigations into user experience. There has been work done to amend existing tools for related technology (e.g., augmented reality) to a VS context; however, these are not widely used in the literature (Azher et al., 2023). While quantitative usability tools such as the SUS are widely used in the instructional design literature due to their broad applicability across many system types (Brooke, 1996), their general nature may not capture the design-specific challenges unique to healthcare-related VS. Indeed, quantitative measures offer valuable objective data, but they may not fully capture the nuanced and context-specific challenges learners encounter in VS environments. Therefore, a mixed-methods approach integrating quantitative measures with qualitative analyses can provide a more comprehensive understanding of the learner experience.

Towards this end, there is little prior work systematically identifying and categorizing these context-specific issues in healthcare-related VSs, despite their potential to directly influence user experience and learning outcomes (Azher et al., 2023). This highlights the need for approaches (e.g., mixed methods) that can capture more granular, simulation-specific usability challenges, which could in turn inform the refinement of existing tools or support the development of VS-specific measures to complement established instruments such as the SUS.

To achieve this, we drew upon the literature to define usability in VS-related contexts (Azher et al., 2024). Specifically, usability in VS is the effectiveness, intuitiveness, and satisfaction with which users can achieve specified goals in interactive environments (Webster & Dues, 2017). Usability is an important aspect to consider as it directly impacts participants’ learning experiences. For example, a student who is unable to find the correct examination option within a VS (poor navigational usability) might consequently feel discouraged in their learning.

### Gap in Virtual Simulation Literature

While the aforementioned studies make important contributions to the literature, the use of non-VS specific usability self-reports raises questions regarding content validity within the VS context (e.g., are these tools *actually* measuring ease of use and usability properly?) (DePoy & Gitlin, 2016). However, no documented validated tools exist that can measure the usability of virtual simulations, specifically, and limited work is present to guide the development of such tools (e.g., which aspects of a virtual simulation are important for user experience).

Another gap in the literature, related to the user experience within a VS, is identifying challenges and/or bothersome events that exist within current VSs. The literature currently does not provide much insight into what specific aspects of VSs may need improvement from a software level. For example, a VS may be generally useable (according to general measurements such as the SUS) but may offer a suboptimal experience due to an abundance of software-related bugs, poor instructions, and other software-level challenges. This gap has been cited in the past as being crucial to investigate to enhance the quality and efficacy of VSs (Azher et al., 2023). This is directly related to the concept of usability as VSs that contain a lot of challenging/bothersome aspects may negatively impact the user experience and thus consequently learning.

As such, we aimed to address these gaps by qualitatively examining participants’ experiences within the same clinical VS across both modalities (screen-based VS and headset VR), drawing upon changes in participants’ physiological arousal levels throughout a VS. This use of physiological arousal adds novel value to the literature as it has been directly linked with the emotional state of an individual (e.g., a frustrated individual would have elevated physiological arousal levels), providing valuable insight into specific experiences within a VS that may be challenging/bothersome (Kazemitabar et al., 2021). This application of physiological arousal measurement via electrodermal activity (EDA) in a VS context to support identification of challenging/bothersome events has not been previously explored in nursing education. Integrating EDA measurements within VSs can provide objective data on the physiological impacts of these simulations, contributing to a more comprehensive understanding of how learners interact with and respond to VS environments. Knowing which parts of a VS are physiologically arousing to students adds both psychological and educational value by identifying potentially *fixable* challenging/bothersome events that are linked to adverse emotional states (e.g., frustration) in a learning context (Novak et al., 2023). These challenging/bothersome events can be addressed in VS development, providing a more optimized experience during learning.

While EDA is an established method for measuring physiological arousal in healthcare simulation (Harley et al., 2019; Moreno et al., 2025), prior research has also linked changes in EDA as a measuring tool to assess the degree of cognitive load and stress experienced in various contexts (Buchwald et al., 2019; Rahma et al., 2022; Romine et al., 2022). For example, Rahma et al. (2022) and Romine et al. (2022) showcase that EDA measurements can be used to identify periods of cognitive stress and high mental effort, respectively. These findings support the use of EDA as a proxy for cognitive processing demands in interactive and educational environments, which aligns with the aims of the present study. Furthermore Buchwald et al. (2019) demonstrated that playing a video game, a cognitively demanding and interactive task, elicited the highest EDA responses compared to both a resting condition and an elevated-breathing condition, with most participants reaching their peak EDA during gameplay. This pattern suggests that elevated EDA in such contexts may also reflect increased cognitive or mental demands. Given that healthcare VSs share similarities with games in requiring rapid decision-making, sustained attention, and interaction with dynamic on-screen content, prior work supports the use of EDA as an indicator of cognitive load in our study’s context.

Relatedly, this approach of combining qualitative and quantitative investigations through different data streams provides a source of convergent validity, reducing subjectivity. For example, to provide evidence of validity for our qualitative identification of challenging/bothersome experiences, we examine physiological arousal levels, which in theory, should be elevated when participants experience challenging/bothersome events within a VS, affirming our accurate coding of “challenging/bothersome” events and thus reducing subjectivity.

The specific goal of this study was to examine specific key challenging/bothersome events or aspects of a VS that students were struggling with, determine if specific challenging/bothersome event categories are more important than others and how these challenging/bothersome events impact previously investigated aspects of students’ performance, ratings of usability, and cognitive load as they interact with a clinical VS. Importantly, this study directly compares these factors between screen-based and headset VR modalities within the same VS scenario and platform, ensuring that scenario content, difficulty, and design features are held constant across modalities. Through identification of these challenging/bothersome events and their associated categories, we stand to help inspire items for the development of a VS-specific usability self-report tool and offer valuable insight for developers, educators, and other stakeholders to consider when evaluating and developing VSs.

## Theoretical Frameworks

### Emotions & Emotional Arousal

To provide evidence of validity for our coding of challenging/bothersome events, we measured participants’ emotional arousal during the VS, drawing on Pekrun’s Control-Value Theory (CVT) of achievement emotions (Pekrun, 2024). CVT posits that emotions vary by valence (positive to negative) and arousal (activating to deactivating) (Pekrun, 2024). Positive activating emotions (e.g., enjoyment) enhance achievement while negative deactivating emotions (e.g., boredom) impair it (Duffy et al., 2020; LeBlanc, 2019). Effects of positive deactivating and negative activating emotions vary based on a multitude of factors (e.g., perceived control and emotional intensity) (Duffy et al., 2020; LeBlanc, 2019; Pekrun, 2024). CVT is especially suited to this study due to its inclusion of arousal and application in health professions education (Azher et al., 2024; Duffy et al., 2020).

This study focused on activation, which can be captured using measurements of physiological arousal (Pekrun, 2024). Electrodermal activity (EDA) is a measure of electrical activity of the skin and an indicator of physiological arousal (Horvers et al., 2021). Skin conductance is a type of EDA measure. Tonic EDA is the continuous skin conductance level, representing overall arousal (Braithwaite et al., 2015; Horvers et al., 2021), while phasic EDA is a measure of stimulus-specific conductance changes and thus a representative of stimulus-specific arousal (Horvers et al., 2021). We focused on phasic EDA as it aligns with our event-based, qualitative approach, allowing us to observe arousal changes tied to specific moments within a VS (Harley et al., 2019). By doing so, we provide supporting evidence that each qualitatively coded challenging/bothersome event is indeed challenging/bothersome as it should be accompanied by elevations in physiological arousal (suggestive of emotions like frustration).

According to CVT, negatively valanced activating emotions such as frustration and anger can arise when learners encounter obstacles that challenge their valued learning goals and when perceived control over the situation is low (Pekrun, 2024). In educational technology contexts, including virtual simulations, such emotions may be triggered by design-related barriers or restrictions. These emotional responses are not merely affective states but are accompanied by physiological changes, such as increased arousal, which can be objectively measured (Buchwald et al., 2019; Pekrun, 2024; Rahma et al., 2022; Romine et al., 2022). Thus the combination of CVT provides a theoretical basis for our use of EDA to interpret learners’ responses to challenging or bothersome events in virtual simulations.

### Cognitive Theory of Multimedia Learning & Cognitive Load Theory

To guide our interpretation of findings, we drew upon Mayer’s cognitive theory of multimedia learning given its applicability to the VS context (Mayer, 2020, 2024). The theory posits that three key assumptions govern multimedia learning: (1) the dual-channel assumption (the presence of separate visual and auditory processing channels); (2) there is a finite capacity for information processing; and (3) active-processing, suggesting that learners engage in active cognitive processes to construct meaningful knowledge (Mayer, 2020, 2024). This framework draws from cognitive load theory, which distinguishes between intrinsic, extrinsic, and germane cognitive load (Leppink et al., 2013). Intrinsic load relates to the inherent demands of learning educational material; extrinsic load regards the presentation of the material; and germane load concerns the effort to organize and understand information (Leppink et al., 2013). We chose this framework over related theories like productive failure (Sinha & Kapur, 2021) due to the high element interactivity of VSs, especially in a medical context (Leppink, 2020). Element interactivity refers to how interconnected the learning components are and how much processing they require (Duran et al., 2022). Previous work has shown cognitive load theory to be applicable in educational settings with high element interactivity (Duran et al., 2022). Thus, given the complex nature of the VS (and thereby high element interactivity), we opted to utilize cognitive load theory to help guide our discussion.

In this study, our primary interest was in extrinsic cognitive load, which refers to the mental effort imposed by the way information or tasks are presented rather than by the inherent complexity of the material (Leppink et al., 2013). Extrinsic load is particularly relevant in VS contexts because interface design, navigation pathways, and system feedback can either reduce or exacerbate the mental resources needed for task completion (Mayer, 2020, 2024). Conversely, intrinsic cognitive load—driven by the inherent difficulty of the learning material—was not the primary focus on this study as our aim was examining design-specific elements of the VS environment that influence usability. As such, we excluded intrinsic cognitive load from the focus of our analysis post-data collection. Additionally, we followed recent shifts in cognitive load literature that reconceptualize germane load as overlapping with other types and thus excluded it (Leppink, 2020). Accordingly, our study emphasizes extrinsic cognitive load, as it is most applicable to novel educational technology and the context of challenging/bothersome events.

## Methods

This study and all procedures encompassed therein followed institutional guidelines and have been approved by an institutional review board (IRB Study Number A11-B87-20B (20-11-022)).

This mixed-methods study sought to answer the following questions:

When nursing students interact with an educational VS with their assigned modality:

RQ1 **(A)** what types of challenging/bothersome events categories are observed? And **(b)** does one modality (VS versus VR) foster more challenging/bothersome events than the other?

RQ2 While accounting for students’ baseline EDA, does nursing students’ emotional arousal statistically significantly change from right before to directly after encountering challenging/bothersome events (regardless of category)?

RQ3 While accounting for students’ baseline EDA, is there a significant change in a challenging/bothersome event category’s EDA from before and after challenging/bothersome events when compared with other categories?

RQ4 While accounting for students’ baseline EDA, does change in a challenging/bothersome event category’s EDA from before and after challenging/bothersome events influence students’ performance, usability, and extrinsic cognitive load relative to other challenging/bothersome event categories?

We acknowledge that there are varying definitions in the literature regarding mixed methods versus multi-methods designs (Anguera et al., 2018; Aspers & Corte, 2019; Creswell, 1999; Steinmetz-Wood et al., 2019). While perspectives differ, we align with definitions that emphasize *integration* of qualitative and quantitative components *within a single study* (Anguera et al., 2018; Creswell, 1999; Steinmetz-Wood et al., 2019).

### Sample

In a mixed-methods study, third-year nursing students (n = 43) from a 4-year program (BScN) were recruited as part of a clinical course in their program from a North American University for a larger study (95.56% participation rate). The control group (n = 14), which did not interact with the VS, was included in the broader study to address separate research questions not relevant to the present manuscript. As such, control group data were excluded from the current analyses. Of the remaining twenty-nine students, twenty students (see Table 1 for relevant demographics) provided consent to have *both* audio-video recordings and EDA data (via Empatica E4 bracelets) used for analysis. The data from these twenty students was used in this manuscript. The present study’s sample size is comparable to previous work that has utilized a multimodal data collection approach in virtual environments (Antoniou et al., 2020; Chiossi et al., 2022; Collins et al., 2019). Given the granular nature of working with such specialized data channels, our sample size is comparable to other studies utilizing similar data (Horvers et al., 2021). See results section for power calculations. All included participants gave informed written consent. Students received a $10 e-gift card for participating.

**Table 1.** Relevant Demographic Information of Participants.

Characteristic	Screen-based virtual Simulation	Headset virtual Reality	Total
	n	n	n
Participants	10	10	20
Gender			
Male	–	–	–
Female	10	10	20
Year of program			
U0 (Year 1)	–	–	–
U1 (Year 2)	–	–	–
U2 (Year 3)	10	10	20
U3 (Year 4)	–	–	–
Weekly gaming hours			
None	8	9	17
1-5 hours/week	–	1	1
6-10 hours/week	1	–	1
20+ hours/week	1	–	1
Previous VS experience			
None	1	2	3
1-5 times	5	1	6
5-10 times	1	3	4
10+ times	3	4	7
Previous VR experience			
None	7	8	15
1-5 times	2	1	3
10+ times	1	1	2

### Study Design

**Figure 1.**
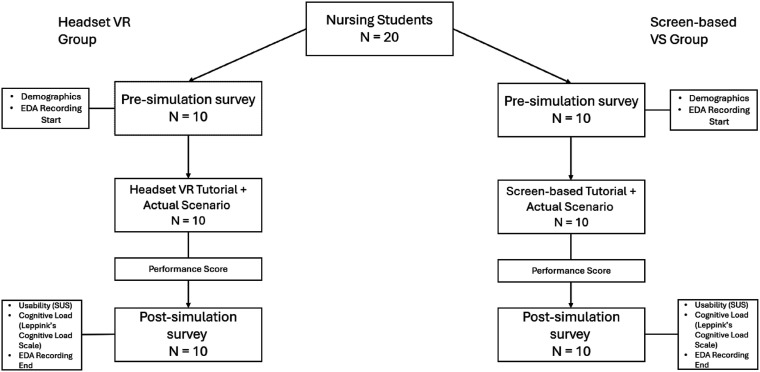
Outline of study design. *Note.* SUS = system usability scale (Brooke, 1996); VR = virtual reality, VS = virtual simulation, EDA = electrodermal activity. Nursing students were randomized into either the headset VR group (left) or the screen-based VS group (right). Both groups completed a pre-simulation survey which captured their general demographic information. During this phase, EDA recordings were started and continued throughout the study session. Upon completion of the pre-simulation survey, students completed a tutorial VS scenario, followed by the real VS scenario. At the end of the scenario, a software-generated performance score was given. A post-simulation survey was then completed which captured students’ perceptions of usability using the system usability scale (Brooke, 1996) and cognitive load using Leppink’s scale (Leppink et al., 2013). After completing the post-simulation survey, the EDA recordings were ended, and participants were free to leave.

Participants (P.Ns.) completed a 2-hour study session that involved a pre-simulation survey, a tutorial simulation, a VS scenario (Maria Perez [SMH001UK]) on the Oxford Medical Simulation (OMS; 2023 version (OMS, London, UK)) platform, and a post-simulation survey (Figure 1). While OMS may be more recognized for its immersive headset VR experience, the platform is intentionally designed to operate equivalently in both headset VR and screen-based desktop formats (Azher et al., 2023; Harley et al., 2023), providing identical case content, objectives, and interactive features across modalities. Thus, all participants engaged with the same simulation scenario, with identical case content, objectives, and difficulty level across both modalities. The only difference between conditions was the modality, either screen-based VS or headset VR. The headset VR group used Meta Quest 2 headsets (Meta Platforms Inc., Menlo Park, CA, USA) for tutorial and scenario simulations, while the screen-based VS group used a laptop with a mouse. The tutorial scenario provided instructions on navigating and interacting within the simulation, and for the VR group, this included specific orientation to the virtual environment to ensure familiarity with the hardware. Prior to beginning the VS scenario, all students received a brief pre-simulation document outlining the setting (an emergency department), and objectives (e.g., assessing the patient, communicating findings and management plan to the most appropriate provider). In the VS scenario, students assumed the role of a nurse managing a patient with symptoms of an acute anxiety attack. They could interact with the patient, use a virtual assistant (fellow nurse), contact other providers (via phone), order labs, and chart findings (via computer) in the simulated environment. The simulation was followed by a verbal debriefing facilitated by the VS software, prompting students to reflect on what went well, what did not, and how they felt during the experience. After completing the scenario, the OMS software generated a final performance score based on technical and non-technical skills.

The tutorial scenario provided instructions on navigating and interacting within the simulation. The VS scenario involved assuming the role of a nurse who was managing a patient with symptoms of an acute anxiety attack in the emergency room. After completing the VS scenario, the OMS software generated a final score to gauge each student’s performance relative to ideal management of the patient. This included examining both technical and non-technical skills exhibited by the student in the VS.

### Survey Instruments

The pre-simulation survey gathered general demographic information regarding participants (e.g., age, gender, visible minority status, etc.). The post-simulation survey was utilized to quantitatively assess students’ general user experience using the system usability scale (Brooke, 1996), and students’ rating of cognitive load types using Leppink’s cognitive load scale (Leppink et al., 2013).

#### System Usability Scale

The SUS is a 5-point Likert scale consisting of 10 items (Brooke, 1996). It requires post-hoc transformations in order to output a final scale between 0-100 (Brooke, 1996). The SUS is a general measure of a system’s usability and there is no official standard for interpreting scores. The SUS has been widely utilized in over 3000 studies and has shown strong evidence of validity (Bangor et al., 2008, 2009). However, results can be interpreted based on recent work, which has aimed to attach grade, and adjectival ratings to different SUS values and ranges (Bangor et al., 2009). This method of interpreting SUS scores has been utilized in VS literature (Azher et al., 2023; Brown et al., 2021; Butt et al., 2018; Harley et al., 2023).

#### Leppink’s Cognitive Load Scale

Leppink’s cognitive load scale is a 10-point Likert scale consisting of 10 items (Leppink et al., 2013). It measures three cognitive load types: intrinsic, extrinsic, and germane load (Leppink et al., 2013). This scale has shown evidence of validity across a series of studies conducted by Leppink (Leppink et al., 2013). This tool is also applicable when measuring cognitive load in non-conventional educational interventions (Azher et al., 2023).

### Qualitative Coding

The lead author conducted thematic analysis on simulation recordings to identify challenging/bothersome events encountered by nursing students. As students were not verbally prompted during the simulation to avoid distraction, there were no verbal responses to transcribe. Analysis followed the six-phase process outlined by Nowell et al. (2017) and based on inductive thematic analysis from Braun and Clarke (2006). Feedback from three practicing nurses and nursing educators on the research team informed theme validation by providing input on the generated themes, their definitions, and how they were applied to the virtual simulation recordings. Their credentials included a registered nurse with a master’s degree in advanced nursing and assistant professor role, a registered nurse with pediatric critical care certification and faculty lecturer role, and a registered nurse with a master’s degree, nurse educator certification, and director of a nursing simulation centre. Their feedback offered a clinical lens to ensure that the themes accurately reflect relevant and meaningful aspects of nursing practice.

Additionally, the senior author reviewed the interpretations throughout the analysis process to reduce bias and ensure conceptual accuracy and helped generate and define themes in alignment with relevant psychological constructs and literature. They also assisted in organizing codes into themes, ensuring that the categorizations reflected established principles in educational and cognitive psychology. The senior author has extensive experience conducting and publishing research in healthcare virtual simulations, educational psychology, and holds a leadership role in a large North American university-affiliated simulation centre.

To enhance consistency, we consulted standardized sources (e.g., average reading speeds) to inform coding decisions (e.g., determining when participants took too long to read a prompt) (Brysbaert, 2019)). Codes were refined and organized into six superordinate themes representing challenging/bothersome event categories. The resulting categories are referenced throughout this paper as “challenging/bothersome event categories”. Furthermore, to establish qualitative rigor, we employed Lincoln & Guba’s (1985) trustworthiness criteria, guided by Ahmed’s (2024) application to health professions education. A detailed assessment of these criteria is available in Supplemental Material 1.

### Electrodermal Activity and Statistical Analysis

EDA was measured using Empatica E4 wristband sensors (Empatica Inc., Cambridge, MA, USA) which show evidence of validity and are reliable measures of physiology across various contexts (Borrego et al., 2019; Horvers et al., 2021; Schuurmans et al., 2020). Participants wore the devices throughout the study, with a 3-minute seated period prior to the tutorial simulation used as a baseline (Horvers et al., 2021). Synchronization between video and EDA data was ensured using the built-in E4 button pressed before and after each simulation.

**Figure 2.**
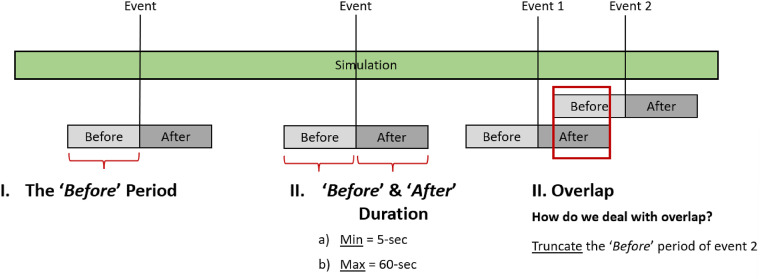
Schematic of thresholds and rules applied to periods of interest for each event. *Note.* The above figure pictorially represents the before period and after period of each challenging/bothersome event (I). The before period and after period were variable in duration, with a minimum time of 5 seconds and a maximum time of 60 seconds (II). In the case of overlap between two events, we truncated the before period of the second event to accommodate capturing the after period from the preceding event (III).

To evaluate arousal related to challenging/bothersome events, we defined distinct “before” and “after” windows for each coded event. These windows captured baseline and reactive EDA patterns, respectively, with durations ranging from 5 to 60 seconds, consistent with established practices (Horvers et al., 2021; Malmberg et al., 2019). Events with overlapping time windows were excluded (*N* = 40), leaving 122 coded events for analysis. A schematic of these thresholds is presented in Figure 2.

**Figure 3.**
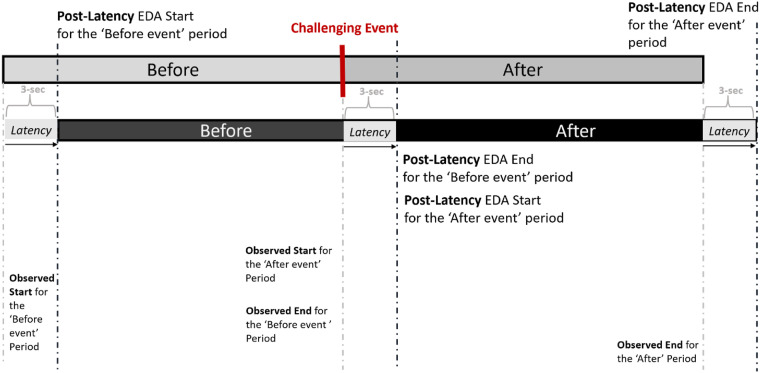
Methodological approach to analyze EDA data from qualitatively coded events. *Note.* This figure outlines the methodology for EDA processing per qualitative event. After qualitative coding of each challenging/bothersome event (illustrated by red line in the figure), average EDA from before and after the event was captured. This led to the generation of two segments of average EDA, the “before period” and the “after period”, per event. Both segments were capped at 60 seconds in accordance with previous literature (Horvers et al., 2021). A 3-second delay was manually added to each segment to account for natural EDA latency in response to stimuli (e.g., the challenging/bothersome event) (Horvers et al., 2021). The latency-adjusted average EDA was subsequently standardized via z-scores to allow comparability between participants (as not all before and after periods were composed of 60 seconds). This yielded average sEDA values for the “before period” and “after period” of each event.

Due to the physiological latency of EDA responses, we applied a 3-second delay to the start of both before and after periods to better capture stimulus-linked arousal (Boucsein, 2012; Caruelle et al., 2019; Sjouwerman & Lonsdorf, 2019). Weighted averages were then computed for each period to accommodate variable window lengths while adhering to the minimum/maximum thresholds. This latency-adjusted approach is visualized in Figure 3. As this was a very detailed process, only a summary is presented in this section. A more detailed explanation of the EDA analysis process can be found in Supplemental Material 2.

EDA data was preprocessed in Ledalab (v3.4.9) in MATLAB, including visual inspection, low-pass filtering (1 Hz), and manual correction of artifacts. Continuous Decomposition Analysis (CDA) was applied to extract phasic EDA, representing stimulus-specific arousal (Chu et al., 2024; Collins et al., 2019). We focused on phasic EDA due to its sensitivity to discrete events, with a threshold of 0.05 µS used for valid responses (Benedek & Kaernbach, 2010a; 2010b). To account for inter-individual variability, phasic EDA values were standardized (sEDA) using z-scores. This term will be utilized from this point forward.

## Results

***RQ1a:*** Six superordinate categories emerged for challenging/bothersome experiences: (1) Software-related restrictions & bugs, (28 instances), (2) Confusion/Lack of success (42 instances), (3) Negative affect (8 instances), (4) Technical errors (31 instances), (5) Neglect of instruction (5 instances), and (6) Other (48 instances). A total of 162 events were coded. Table 2 provides a detailed visual representation and written description of the categories and their subordinate branches while a general description of each category is given in Table 3**.** See Supplemental Material 3 for a breakdown by participant.

**Table 2.** Detailed Qualitative Coding Results for Challenging/Bothersome Event Categories.

Superordinate categories	Definition	Examples	Subordinate level 1	Definition	Subordinate level 2	Definition	Examples of codes
1. Software-related restrictions & bugs	A flaw, error, or fault in the operation of the software that produces an incorrect or unexpected result	Participant is unable to access the completed anxiety score card on the computer as it will not appear until the nurse dialogue ends (PN 10)	1.1. Scorecards and tests	Bugs relating to different medical tests and scorecards			Participant is unable to access the completed suicide score card on the computer as it will not appear until the nurse dialogue ends (PN 13, example of software-related restriction (software wants to prevent students from moving too fast))
			1.2. Overlapping pop-ups	Bugs relating to overlapping interfaces			Participant clicks the depression scorecard on the computer and ECG results pop up on top of the depression scorecard (PN 16, example of software-related bug)
			1.3. Lack of pop-ups	Bugs relating to lack of interface after interaction			Participant clicks medication list option but nothing pops up in response (PN 40, example of software-related restriction (software wants to prevent students from moving too fast))
			1.4. Other	General bugs			Participant is unable to interact with the computer after having exited the medical cabinet (PN 31)
2. Confusion/Lack of success	Difficulty trying to understand what is currently going on and/or how to proceed within the virtual simulation	Participant reviews most body systems one by one (could indicate that they do not know next steps) (PN 7)	2.1. Due to participant’s knowledge	Confusion that stems from the participant own knowledge of the simulation	2.1.1. Conceptual knowledge	Knowledge of classification, categories, principles, generalizations, theories, models, and structures	Participant blankly stares at patient for 16 seconds without making any action (participant looks frozen in confusion) (PN 4)
					2.1.2. Procedural knowledge	Knowledge of subject-specific skills, algorithms, techniques, and criteria for determining when to use appropriate procedures	Participant keeps switching back and forth between medication list and medicine cabinet without picking an option for one minute (could indicate lack of direction) (PN 19)
			2.2. Due to software-related reason(s)	Confusion that stems from software-related elements	2.2.1. Virtual agent communication	Confusion involving the nurse assistant or phone-based assistants	[Participant calls social worker on the phone] after hearing their response, participant raises eyebrow on one eye and rocks head left and right (PN 24)
					2.2.2. Patient communication	Confusion involving communication with the virtual patient	Participant clicks “provide reassurance” and upon hearing the response, scrunches her eyebrows, squints eyes, and tilts head towards the left (this is likely because the spoken sentence does not sound like reassurance) (PN 16)
					2.2.3. Software design	Confusing involving the design of the software	Participant asks researchers what can she do if medication cabinet dosage does not match with the patient chart [leads to confusion between participant and researchers] (PN 30)
3. Negative affect	Feeling visibly upset or irritated due to the inability to achieve a desired outcome	Participant lets out a loud “sigh” after consistently encountering a bug and holds her forehead (PN 31)					
4. Technical errors	Unintentional actions or errors caused by the participant that were not supposed to be conducted	Participant measures the respiratory rate and in unable to enter it into the EWS because she has already submitted and cannot edit (PN 4)					
5. Neglect of instruction	Intentional actions that go against the instructions that are being provided	Participant attempts to click “next” after only choosing one option during the SBAR prompt [the prompt explicitly asks her to pick three] (PN 22)					
6. Other	General observations that do not fall under the above categories	Participant tends to click outside the box instead of clicking “close” (PN 1)					

*Note.* The aforementioned table outlines the super and subordinate themes/categories that were formulated from the negative codes after qualitative analysis of the audio-video recordings. Example codes with square parenthesis indicate actions/facts within the simulation recording while rounded parentheses indicate the researcher’s thoughts. The superordinate category of *Software-related Restrictions & Bugs* included both intentional restrictions (which were inferred to be perceived as bugs by students) and unintentional software-related bugs. Examples of both restrictions and bugs can be seen in the “Example of Codes” section for superordinate category 1.

**Table 3.** General Description of Each Challenging/Bothersome Event Category.

Challenging/Bothersome Event Category	Description
Software-related restrictions & bugs	This category was developed through observations of flaws and errors within the VS software which produced incorrect or unexpected results
Confusion/lack of success	This category was formulated due to a collection of codes that suggested that participants were having a challenging time understanding the VS in general and/or did not know how to progress
Negative affect	This category was formulated due to observations where participants were visibly upset and/or irritated due to their inability to achieve a desired outcome
Technical errors	This category consisted of codes where participants made errors
Neglect of instruction	This category contained codes where participants were explicitly going against instructions that were provided to them by the software
Other	This category contained codes that could not be categorized into any of the other superordinate categories

***RQ1b*:** Overall, **across all events**, screen-based VS yielded 92 (56.79%) instances of challenging/bothersome events while headset VR yielded 70 (43.21%). Next, **looking specifically at each category**, *Confusion/lack of success* had 18 instances (42.86%) for screen-based VS and 24 (57.14%) for headset VR. *Technical errors* had 18 (58.06%) instances for screen-based VS and 13 (41.94%) for headset VR. S*oftware-related restrictions & bugs* had 20 instances (71.43%) for screen-based VS and 8 (28.57%) for headset VR. Furthermore, *negative affect* had 8 (100%) instances for screen-based VS and 0 for headset VR. *Neglect of instruction* had 3 (60%) for screen-based VS and 2 (20%) for headset VR. Lastly, the *Other* category contained 25 (52.08%) instances for screen-based VS and 23 (47.92%) for headset VR. Results are summarized in Table 4. Importantly, within *Software-related restrictions & bugs*, several observations indicated that participants attempted actions (e.g., accessing test results before patient dialogue completion) that were intentionally restricted by the system. In these cases, participants often re-attempted the same action or expressed visible signs of frustration, suggesting they interpreted the intentional restriction as bugs.

**Table 4.** Frequencies and Percentages of Coded Challenging/Bothersome Events.

Challenging/bothersome event categories	Frequencies		
	Screen-based VS	Headset VR	Total
1. Software-related restrictions & bugs	20 (71.43%)	8 (28.57%)	28
1.1. Scorecards and tests	9 (81.82%)	2 (18.18%)	11
1.2. Overlapping pop-ups	6 (85.71%)	1 (14.29%)	7
1.3. Lack of pop-ups	4 (80%)	1 (20%)	5
1.4. Other	1 (20%)	4 (80%)	5
2. Confusion/Lack of success	18 (42.86%)	24 (57.14%)	42
2.1. Due to participant’s knowledge	8 (44.44%)	10 (55.56%)	18
2.1.1. Conceptual knowledge	2 (33.33%)	4 (66.67%)	6
2.1.2. Procedurak Knowledge	6 (50%)	6 (50%)	12
2.2. Due to software-related reasons	10 (41.67%)	14 (58.33%)	24
2.2.1. Virtual agent communication	6 (75%)	2 (25%)	8
2.2.2. Patient communication	2 (50%)	2 (50%)	4
2.2.3. Software design	2 (16.66%)	10 (83.34%)	12
3. Negative affect	8 (100%)	0	8
4. Technical errors	18 (58.06%)	13 (41.94%)	31
5. Neglect of instruction	3 (60%)	2 (40%)	5
6. Other	25 (52.08%)	23 (47.92%)	48
Overall	92 (56.79%)	70 (43.21%)	162

*Note.* The above table contains the frequencies and percentages of coded challenging/bothersome events based on superordinate and subordinate themes/categories. The frequencies are listed with accompanying percentages (relative to the total number of codes in that category) in parentheses.

***RQ2:*** A repeated measures ANCOVA with a Greenhouse-Geisser correction was used to compare sEDA before and after challenging/bothersome events with baseline sEDA as a covariate. There was a statistically significant effect of time on sEDA, *F*(1,18) = 6.31, *p* = 0.02, $\eta_{p}^{2}$ = 0.26, observed power = 0.66. Pairwise comparisons revealed that participants’ post-challenging/bothersome event sEDA (*M* = 0.02, *SE* = 0.07) was significantly higher (*p* = 0.03, 95% CI [0.01, 0.14]) than their pre-challenging/bothersome event sEDA (*M* = −0.05, *SE* = 0.06).

***RQ3:*** Multiple linear regression with cluster robust errors was conducted to compare sEDA change from pre-event to post-event between event categories. As there were low numbers of observations in the *Negative Affect*, and *Neglect of Instruction* categories, they were excluded from this analysis to achieve adequate power. The *Other* category was also removed as it contained codes that could not congregate to generate a broader category. This left: *Software-related Restrictions & Bugs*, *Confusion/Lack of Success*, and *Technical Errors* for analysis. An analysis which includes all challenging/bothersome event categories can be found in Supplemental Material 4. G*Power (version 3.1.9.7) estimated *a priori* sample size of 24 (adjusted α = .017, power = 0.8, effect = 0.8, predictors = 3); 44 observations were analyzed, superseding minimum requirements. The analysis revealed no significant differences between event categories, *F*(3, 19) = 1.18, *p* = 0.34, R^2^ = 0.05, *post-hoc* power = 0.09. Results are summarized in Table 5.

**Table 5.** Multiple Linear Regression With Cluster Robust Errors: Examining Changes in sEDA Across Event Categories.

Predictor variables	*b*	Robust *SE*	*t*	*p*	95% CI	
					*LL*	*UL*
Baseline sEDA	−0.06	0.06	−0.92	0.37	−0.19	0.08
Intercept	0.05	0.10	0.47	0.64	−0.16	0.25
Software-related restrictions & bugs sEDA change	Ref	Ref	Ref	Ref	Ref	Ref
Confusion/Lack of success sEDA change	0.12	0.11	1.16	0.26	−0.10	0.34
Technical errors sEDA change	−0.09	0.24	−0.36	0.72	−0.59	0.42

*Note.* N = 44; R^2^ = 0.05; Root-mean-square deviation = 0.46; *b = coefficient;* Robust *SE* = Robust standard error; CI = confident interval; *LL* = lower limit: *UL* = upper limit. Ref indicates the reference category to which comparisons were made. Intercept indicates the predicted change in sEDA for the reference event category when all other predictors are held at zero. Software-related restrictions & bugs included flaws, errors, or faults in the operation of the VS that produced an incorrect or unexpected result; Confusion/lack of success was regarded as difficulty trying to understand what was going on and/or how to proceed within the VS; Technical errors included unintentional actions or errors that were not supposed to be conducted; All variations of the above comparison (using each category as a reference point) were run and yielded non-significant results.

***RQ4:*** The same exclusions and sample size calculations applied as RQ3. The remaining categories were input into a multiple linear regression with cluster robust errors to examine if the change in a category’s EDA from before and after challenging/bothersome events influences students’ performance, usability, and extrinsic cognitive load. An analysis which includes all challenging/bothersome event categories for this research question can be found in Supplemental Material 5 for performance, usability, and extrinsic cognitive load. The analysis revealed that for performance and usability, there were no significant results. However, for extrinsic cognitive load, the model was significant, *F*(6, 19) = 3.81, *p* = 0.01, R^2^ = 0.10, *post-hoc* power = 0.24. Furthermore, it was found that as *overall* EDA change (from before challenging/bothersome event to after) increased, extrinsic cognitive load significantly increased (*b =* 1.05, *RSE =* 0.40, *t* = 2.670, *p =* 0.02, η^2^ = 0.07, *post-hoc* power = 0.83). Pairwise comparisons (adjusted α = 0.02) showcased that the EDA change for the *Technical Errors* category significantly negatively predicts extrinsic cognitive load compared to the EDA change associated with *Software-related restrictions & bugs* (*b = -*1.11, *RSE =* 0.37, *t* = −3.00, *p =* 0.01, η^2^ = 0.17, *post-hoc* power = 0.98). Results are summarized in Tables 6.

**Table 6.** Multiple Linear Regression With Cluster Robust Errors: Examining if Change in a Category’s EDA From Before and After Challenging/Bothersome Events Influences Students’ Rating of Performance, Usability, or Extrinsic Cognitive Load Relative to Other Event Categories.

Performance score	*b*	Robust *SE*	*t*	*p*	95% CI	
					*LL*	*UL*
Change in category EDA	12.52	13.29	0.94	0.36	−15.31	40.35
Software-related restrictions & bugs sEDA change	Ref	Ref	Ref	Ref	Ref	Ref
Confusion/Lack of success sEDA change	−12.93	19.18	−0.67	0.51	−53.07	27.20
Technical errors sEDA change	−21.02	15.10	−1.39	0.18	−52.64	10.59
Baseline sEDA	−3.24	5.23	−0.62	0.54	−14.19	7.72
Constant	62.90	6.22	10.12	<0.00	49.88	75.91

Pairwise comparisons	*b*	Robust *SE*	*t*	*p*	95% CI	
					*LL*	*UL*
Confusion/Lack of success sEDA change vs software-related restrictions & bugs sEDA change	−12.93	19.18	−0.67	0.51	−53.07	27.20
Technical errors sEDA change vs software-related restrictions & bugs sEDA change	−21.02	15.10	−1.39	0.18	−52.64	10.59
Technical errors sEDA change vs confusion/Lack of success sEDA change	−8.09	11.02	−0.73	0.47	−31.17	14.98

Usability	*b*	Robust *SE*	*t*	*p*	95% CI	
					*LL*	*UL*
Change in category EDA	4.31	8.25	0.52	0.61	−12.96	21.57
Software-related restrictions & bugs sEDA change	Ref	Ref	Ref	Ref	Ref	Ref
Confusion/Lack of success sEDA change	−6.58	15.33	−0.43	0.67	−38.67	25.52
Technical errors sEDA change	−6.21	11.17	−0.56	0.59	−29.59	17.16
Baseline sEDA	1.72	6.23	0.28	0.79	−11.33	14.77
Constant	65.30	6.61	9.87	<0.00	51.46	79.15

Pairwise comparisons	*b*	Robust *SE*	*t*	*p*	95% CI	
					*LL*	*UL*
Confusion/Lack of success sEDA change vs software-related restrictions & bugs sEDA change	−6.58	15.33	−0.43	0.67	−38.67	25.52
Technical errors sEDA change vs software-related restrictions & bugs sEDA change	−6.21	11.17	−0.56	0.59	−29.59	17.16
Technical errors sEDA change vs confusion/Lack of success sEDA change	0.36	11.98	0.03	0.98	−24.71	25.44

Extrinsic cognitive load	*b*	Robust *SE*	*t*	*p*	95% CI	
					*LL*	*UL*
Change in category EDA	1.05	0.40	2.67	0.02	0.23	1.88
Software-related restrictions & bugs sEDA change	Ref	Ref	Ref	Ref	Ref	Ref
Confusion/Lack of success sEDA change	0.08	1.00	0.08	0.93	−2.01	2.18
Technical errors sEDA change	−1.11	0.37	−3.00	0.01	−1.88	−0.34
Baseline sEDA	0.17	0.35	0.48	0.64	−0.56	0.89
Constant	2.00	0.28	7.28	<0.00	1.43	2.58

Pairwise comparisons	*b*	Robust *SE*	*t*	*p*	95% CI	
					*LL*	*UL*
Confusion/Lack of success sEDA change vs software-related restrictions & bugs sEDA change	0.08	1.00	0.08	0.93	−2.01	2.18
Technical errors sEDA change vs software-related restrictions & bugs sEDA change	−1.11	0.37	−3.00	0.01	−1.88	−0.34
Technical errors sEDA change vs confusion/Lack of success sEDA change	−1.19	0.99	−1.21	0.24	−3.25	0.87

*Note.* N = 44 for all models. Performance: R^2^ = 0.08; Root-mean-square deviation = 15.85; F(6, 19) = 1.79, p = 0.15. Usability: R^2^ = 0.05; Root-mean-square deviation = 14.79; F(6, 19) = 0.65, p = 0.69. Extrinsic Cognitive Load: R^2^ = 0.10; Root-mean-square deviation = 1.07; F(6, 19) = 3.81, p = 0.01.

b = coefficient; Robust SE = robust standard error; CI = confidence interval; LL = lower limit; UL = upper limit. “Ref” indicates the reference category used in pairwise comparisons.

## Discussion

### Categorization of Challenges/Bothersome Events

One of the aims of this study was to identify and categorize the types of challenges and bothersome events nursing students face while interacting with a clinical VS. These experiences could be grouped into six main domains: *Software-related restrictions & bugs*, *Confusion/lack of success*, *Negative affect*, *Technical errors*, *Neglect of instruction*, and *Other*. On average, participants encountered ∼6 challenging/bothersome events, reflecting a low frequency. Our findings and subsequent discussion do *not* suggest that the OMS software is problematic but rather identify events in VSs, their impact on physiological arousal, and propose mitigation strategies. This extends prior research (e.g., Azher et al., 2023; Brown et al., 2021; Butt et al., 2018) by moving beyond general usability concerns to provide a structured framework of specific problem categories. These findings support our broader aim to improve virtual simulation usability.

Firstly, *confusion/lack of success* was the second most prominent category (we will talk about most prominent category “*other*” below) and included knowledge and software-related sources. While participant-specific knowledge gaps are important to address in an educational environment, they are hard to generalize, especially due to the heterogeneity between learners across institutions. As such, we focus on software-related causes that can be addressed in future VS design. Codes in this category highlight the need for realistic dialogue, editable inputs (e.g., vital sign documentation), and visible confirmation of user input (e.g., confirming that vital signs on a monitor have cycled). These codes were relatively equally distributed between the two modalities (screen-based VS & headset VR). A similar discussion can be drawn from the *Technical errors* category as most of the codes dealt with potentially confusing VS menus. For example, it was noted that participants kept confusing multiple patient-related menus, which were accessed by clicking different parts of the patient (e.g., clicking the head to talk to the patient, but the body to examine the patient). We note that a singular menu branching into multiple menus could help mitigate these errors. Relatedly, the VS used vertically cascading windows (e.g., multiple options are present in a vertical list) to manage various options available to students. Bernard et al.’s (2003) work showcases visual examples of menu types and examines them through both objective and subjective measures. They showed that cascading menu designs are non-optimal for task completion times when users want to find an explicitly given option (e.g., the physician telling the student to order a urine test) or an implicitly given option (searching for the appropriate test based on patient symptoms) (Bernard et al., 2003). Instead, index-based menu layouts (where all options are presented in an organized manner), despite seeming to overload students with information, are preferred, result in less time to find options, and are equally easy to navigate (Bernard et al., 2003). Thus, future iterations of VS software should consider utilizing index-style menus when possible. Notably, as the OMS software was identical in design between the two modalities, it is not surprising that there were no stark modality-based differences observed.

### Implications for Software Design

*Software-related restrictions and bugs*, and *neglect of instruction* were descriptively skewed towards screen-based VS while *negative affect* was exclusively to screen-based VS. As simulation content was identical between modalities, limited VR experience (as indicated by the pre-simulation survey) may have led students to navigate the VS slower and more cautiously. Consequently, the faster pace of those using screen-based VS may have led to encountering more system restrictions and bugs. This hypothesis has support as one of the primary codes in this challenging/bothersome event category is the observation that students kept wanting to access test results before the patient dialogue ended. Notably, while the superordinate category of *software-related restrictions and bugs* included both *intentional* restrictions and unintentional bugs, as observed in our qualitative data, participants encountering these intentional restrictions often re-attempted the blocked action or displayed frustration, suggesting they perceived them as bugs rather than purposeful design features. Such restrictions should be made clear within the VS’s tutorial and/or introduction scenarios as not to unnecessarily frustrate students. Next, *neglect of instruction*, while skewed towards screen-based VS only contained 5 observations. Given the small number of observations for this category, it is indicative of a positive attribute of the utilized VS software—providing good instructions to students. Lastly, the absence of *negative affect* in headset VR may be attributed to the difficulty in identifying participants’ expressions, unlike in screen-based VS where participants’ faces and hands are fully visible.

Lastly, the *other* category, nearly equally distributed between the two modalities, contained the highest frequency (n = 48) of challenging/bothersome events. Notably, 42 of these instances were the observation of students not letting subtitles from a previous prompt finish prior to clicking on another prompt. It can be inferred that students preferred reading subtitles over listening to the dialogue within the VS. This may be due to the VS containing speech audio that resembled the pace of real-life conversation, which can be slow. This proposed explanation is consistent with Mayer’s cognitive theory of multimedia learning, which suggests that in educational settings, redundancy across information channels (e.g., audio channel or visual channel) should be avoided (Mayer, 2020, 2024). It stands to reason that multiple information channels relaying the same content may result in an increase in extrinsic cognitive load which is detrimental to learning (Leppink et al., 2013). Thus, educators using VS platforms may consider prioritizing one information channel. For VS developers, this may mean incorporating toggle-able features that allow users to reduce information redundancies (e.g., enable/disable subtitles). While for educators, this could take the form of making students aware that such redundancies can be toggled on or off (some students may prefer subtitles *and* audio in cases where the primary language of the VS is not the students’ first language). This reinforces the importance of learner-centered VS design as emphasized in usability literature (Radianti et al., 2020; Webster & Dues, 2017) and aligns with our study’s goal of uncovering specific simulation design elements that impact user experience.

### Convergent Validity for Qualitative Findings

Our qualitative findings identify VS design elements where students struggle, an important goal of our work. This advances the literature as we link these struggles to usability-related design elements which impact the effectiveness and satisfaction of the VS experience explored generally (via quantitative measures only) by previous work (Azher et al., 2023; Brown et al., 2021; Harley et al., 2023). By conducting such a granular qualitative analysis, we help uncover the specific and *fixable* problems within VSs, providing deeper insights into VS design considerations, all while addressing a gap in the literature. Furthermore, our qualitative coding serves as a valuable foundation for the development of tools aimed at measuring user experience in VS-specific contexts, addressing another gap in literature. The identified *superordinate* challenging/bothersome event categories can guide the development of general items for these tools while the *subordinate* categories provide a more focused lens to refine and specify item content. Additionally, comparing modalities allows us to determine which aspects of the VS need emphasis for a particular modality, especially if modality-specific usability tools are developed.

This study also aimed to support the validity of our coding. Indeed, our analysis of students’ EDA surrounding these challenging/bothersome events provides convergent validity for our qualitative work. Specifically, given that students’ physiological arousal significantly increases after encountering challenges/bothersome events, we can reasonably assume that the events were correctly coded as being *challenging/bothersome* experiences for students. Although underpowered, effect sizes were large, offering tentative support for our coding approach. As such, while we interpret our EDA results with cautious optimism, we acknowledge that they also provide tentative validity evidence for our qualitative coding.

### EDA and Its Implications for VS Design

Next, a key goal of this research was to investigate how changes in physiological arousal during different types of challenges relate to students’ experiences, particularly in terms of performance, usability, and cognitive load—filling a gap previously identified in VS literature (Azher et al., 2023). By doing so, we contribute to the literature on the use of EDA in nursing education, specifically in the context of VSs. The application of EDA to evaluate students’ experiences in VSs, especially in nursing education, is unexplored. Previous work has been done in nursing education which draws upon self-report measures (Azher et al., 2023; Harley et al., 2023) or other proxy measures (e.g., pulse rate via a pulse oximeter) (Gutiérrez-Fernández et al., 2024; Ko & Kim, 2022) to infer arousal levels, but none that use EDA in a VS context. Furthermore, to the author’s knowledge, no work exists which utilizes EDA measures to guide interpretations of qualitative findings in nursing education, thereby adding novel value to the literature.

Our EDA findings are particularly insightful when viewed through the theoretical framework of CVT. In our context, it is reasonable to assume that the challenges/bothersome events identified in our qualitative coding elicited *negatively valanced activating* emotions (e.g., frustration) (Pekrun, 2024). This may be due to the lack of control over the challenges/bothersome events encountered by students (e.g., software-related restrictions and bugs), especially in a high-value task (e.g., trying a novel VS). CVT would posit that increases in physiological arousal indicate that students may be experiencing *activating* emotions, and given the challenge/bothersome context, these emotions are likely *negatively valanced* (e.g., anger, frustration), as students may perceive these events as obstacles to their learning goals (high value) (Duffy et al., 2020; Pekrun, 2024). Furthermore, CVT emphasizes the importance of perceived control in the learning process (Pekrun, 2024). The challenging/bothersome events identified in our study may have disrupted students’ sense of control within the VS, especially in cases where they were unintended software-related bugs, thereby eliciting a physiological response, a theory supported by our EDA data. Additional literature on EDA has shown that, when measured using skin conductance, heightened EDA is directly linked to challenging/bothersome stimuli and is indicative of heightened physiological arousal and stress (Chu et al., 2024).

While these challenging/bothersome events significantly increased students’ physiological arousal, when grouped by their respective categories and compared to each other, this significance disappears. This suggests that one challenging/bothersome event category is not necessarily more important to consider than the others in terms of stimulating physiological arousal. Therefore, all categories of challenges/bothersome events are equally important when considering their impact on EDA. This has implications for VS developers, indicating that holistic improvements across all aspects of a VS are needed to decrease challenges/bothersome events encountered by students. Though it should be noted that this analysis was underpowered so results should be interpreted with caution. Given a larger sample size, it stands to reason that one challenging/bothersome event category may become more important to consider than others.

Upon examining other relevant variables in VS education such as performance, usability, and extrinsic cognitive load, we found that the challenging/bothersome event categories are unable to significantly predict performance and usability relative to each other. This shows that challenging/bothersome events need to be addressed by VS developers when optimizing usability of software as well as students’ generated scores, irrespective of the challenging/bothersome events’ classification. However, when attempting to decrease extrinsic cognitive load, special attention to technical errors and software-related restrictions and bugs is required. Specifically, students’ arousal changes from *technical errors* significantly negatively predicted extrinsic cognitive load compared to the arousal change associated with *software-related restrictions and bugs*. This implies that as software-related restrictions and bugs get more arousing (possibly reflecting frustration/confusion), they significantly increase extrinsic cognitive load when compared to the arousal levels induced by technical errors. Given that extrinsic cognitive load is associated with the presentation of educational material and thus important to minimize, it is imperative that VS developers work to minimize these software-related restrictions and bugs. Notably, an elevated cognitive load is not inherently detrimental as complex, authentic learning tasks naturally require higher cognitive processing demands (Leppink et al., 2013; Sweller et al., 2019). However, when excessive load arises from suboptimal interface design or unnecessary task demands (i.e., extrinsic cognitive load), it becomes counterproductive and may hinder learning effectiveness. As such, while we call for holistic improvements across all challenging/bothersome event categories, we draw special attention to reducing software-related restrictions and bugs as they are directly implicated in increasing extrinsic cognitive load. Overall, such insights advance prior work by offering a layered, multimodal understanding of how specific simulation design elements shape learner outcomes—thereby informing both theoretical and practical advancements in VS-based education.

## Strengths, Limitations and Future Directions

Our work is not without limitations. Firstly, only 20 participants were analyzed. Although we met *a priori* sample requirements, some analyses (RQ2 and RQ3) were underpowered. However, large effect sizes in some results (RQ2) support the robustness of findings. Our sample size is comparable to, or exceeds related multimodal studies (Antoniou et al., 2020; Chiossi et al., 2022; Collins et al., 2019). Combined with our granular methodology, this is a notable strength. Our unique approach captures rich learner experience and offers novel contribution to the literature. Despite this, future work should aim to replicate this study with a larger sample to increase power and ensure more reliable detection of effect size.

Furthermore, to address power considerations, we excluded categories with few (less than 10) observations (note: the *other* category was removed due to its lack of ability to form a coherent superordinate category). While non-ideal, this prioritizes statistical integrity, underscoring the rigorous and cautious approach we undertook in our analysis. Future work should expand sample sizes to include these excluded categories.

Additionally, our qualitative coding was based on video inspection, which is inherently subjective. A possible supplement is eye-tracking to pinpoint attention during difficult moments—an approach used in similar research, albeit not examining challenging/bothersome experiences in VSs (Ahn & Harley, 2020; Anbro et al., 2020; Dubovi, 2022). Another limitation of our work is generalizability. Our analysis focused on a specific scenario in a single version of OMS. As such, the simulation itself, including its case content, interface, and level of realism, may have influenced how participants perceived and responded to challenging events. Later versions may address the challenges/bothersome events we identified with our granular analyses and may have different patterns of events in different scenarios. Further, while OMS is a commonly used software in medical education (Azher et al., 2023; Brown et al., 2021, 2023; Harley et al., 2023; Mallik et al., 2021) other software such as UbiSim also exist (Lemée et al., 2024; Zhou et al., 2022). Therefore, our findings and recommendations should be taken with caution when applying across different VS platforms. However, this study pioneers the examination of challenges within VSs, providing direction for exploring challenges in different systems. Future research should extend this work by investigating challenges across VS platforms and scenarios.

Notably, the experience of nursing students may be different from that of medical students, medical residents, and even dentistry students. As such, similar studies are needed in other educational domains to extend findings and examine if similar challenging/bothersome event categories exist for other health professions students. Furthermore, it is important to note that this was a single-institution study, and that cultures of nursing education differ depending on the institution and the resources available. For example, some nursing education institutes may not have access to VS equipment thereby limiting students’ exposure to these technologies. Accordingly, the students may not have the same experiences as those in our study, potentially leading to different results. Future work should replicate the present study across other institutions to examine if different institutional factors influence findings. Lastly, future research should take our qualitative findings as a guiding post in the development of VS-specific usability measurements. By drawing upon our identified superordinate and subordinate categories, future work can develop questionnaire items that target specific aspects of VSs, a gap in the current literature.

Lastly, while this study directly compared screen-based and headset VR modalities within the same simulation scenario, no statistically significant modality-based differences were detected across key outcomes. This limited finding may be due to the small sample size and should be interpreted with caution. Future research with larger and more diverse cohorts is warranted to further examine potential modality effects.

## Conclusions

Overall, our work addresses multiple gaps and provides preliminary evidence in the literature for the importance of addressing challenging/bothersome events in virtual simulations across modalities in nursing education—a gap identified by previous literature (Azher et al., 2024). Our work also drew attention to key challenging/bothersome events encountered by nursing students and categorizes them through our qualitative analysis. These challenging/bothersome categories offer a detailed and robust foundation for the development of VS-specific usability tools that should aim to capture students’ user experience—another existing literature gap. Our subsequent physiological arousal examination suggests there is convergent validity for our qualitative analysis and provides preliminary support for our categorization of challenging/bothersome events and categories—a novel synergistic application of EDA and qualitative coding. Our quantitative additions further suggest that an approach that prioritizes the mitigation of challenging/bothersome events overall is better than an approach that targets specific categories when the goal is to decrease physiological arousal changes. Furthermore, we highlight comparisons between the generated challenging/bothersome event categories in their ability to predict students’ performance, usability, and extrinsic cognitive load, three important variables for VS developers and educators (Azher et al., 2023). We show that a particular emphasis should be placed on reducing software-related restrictions and bugs given their association with increasing extrinsic cognitive load.

These insights should be interpreted as preliminary but promising, as they inform the refinement of current simulation development but also pave the way for future research and development aimed at optimizing virtual learning environments for nursing education. This work adds meaningful value by guiding the creation of VS-specific usability assessment tools, ensuring proper simulator validation, and enhancing the overall effectiveness and user-friendliness of educational VSs.

## Supplemental Material

Supplemental Material - Mixed Methods Examination of Challenging and Bothersome Events in Nursing Virtual Simulations: Comparing Screen-Based and Headset VR Modalities Supplemental Material for Mixed Methods Examination of Challenging and Bothersome Events in Nursing Virtual Simulations: Comparing Screen-Based and Headset VR Modalities by Sayed Azher, Keerat Grewal, Negar Matin, Amanda Cervantes, Caroline Marchionni, Hugo Marchand, Jason M. Harley in Simulation & Gaming.

Supplemental Material - Mixed Methods Examination of Challenging and Bothersome Events in Nursing Virtual Simulations: Comparing Screen-Based and Headset VR Modalities Supplemental Material for Mixed Methods Examination of Challenging and Bothersome Events in Nursing Virtual Simulations: Comparing Screen-Based and Headset VR Modalities by Sayed Azher, Keerat Grewal, Negar Matin, Amanda Cervantes, Caroline Marchionni, Hugo Marchand, Jason M. Harley in Simulation & Gaming.

## Data Availability

The participants of this study did not consent to having their data shared publicly and are from a specialized professional population. As such, due to privacy concerns, data will not be made available.
