# Supplementary material for: Mixed Methods Examination of Challenging and Bothersome Events in Nursing Virtual Simulations: Comparing Screen-Based and Headset VR Modalities
Source: Simul Gaming. 2025 Nov 25;57(4):494–530. doi: 10.1177/10468781251401059 (PMC13298862; doi:10.1177/10468781251401059)
Supplement: Supplemental Material - Mixed Methods Examination of Challenging and Bothersome Events in Nursing Virtual Simulations: Comparing Screen-Based and Headset VR Modalities [file sj-pdf-1-sag-10.1177_10468781251401059.pdf]

Supplemental Material 1

Assessment of Pillars of Qualitative Trustworthiness.

Trustworthiness Component	Component Requirement Met	Explanation
**Credibility**	**Triangulation:** Employ multiple data sources or methods (e.g., interviews, observations, documents) to cross-verify findings.	The authors utilized both qualitative coding and quantitative physiological sensor data to cross-verify findings
**Transferability**	**Thick description:** Provide detailed contextual information to enable readers to assess the transferability of findings.	The authors provide contextual information regarding the virtual simulation, setting of the study, and other relevant aspects.
	**Sampling strategies:** Clearly articulate the sampling process and criteria to justify the potential transferability of the findings.	The authors clearly document the sampling process and criteria in the methodology sections
**Dependability**	**Methodological documentation:** Detail the research procedures and decisions made during the study.	The authors detail the research procedures and decisions extensively in their methodology and results sections
	**Audit trails:** Keep an audit trail of research decisions, changes, and data analysis processes to ensure traceability	An audit trail was kept documenting each decision and change made to both the methodology and result.
**Confirmability**	**Peer debriefing:** Engage with colleagues or experts to review interpretations and findings, minimizing researcher bias.	The lead author worked very closely with the senior author who is an expert in virtual simulations in healthcare and health professions education. The lead author also worked very closely with co-authors that are practicing nurses and nursing educators to minimize researcher bias.

Note. The above table is an adaptation from Ahmed (2024, see References, licensed under a CC-BY 4.0 license) which draws upon seminal work from Lincoln & Guba (1985).

Supplemental Material 2

Detailed Information Regarding Electrodermal Activity and The Associated Statistical Analysis

EDA was measured using Empatica E4 wristband sensors (Empatica Inc., Cambridge, MA, USA). While many sensors exist to measure EDA, we elected to use Empatica E4 due to the high evidence of validity across multiple studies, as well their documented reliability across a range of populations and experimental conditions (Borrego et al., 2019; Garbarino et al., 2014; Horvers et al., 2021; Schuurmans et al., 2020). Electrical conductance across the skin was recorded in microsiemens (μS) at a sampling rate of 4 Hz (2021). Participants who consented to the recording of their EDA signals were instructed to wear the E4 devices throughout the full length of the study. The initial 3-minute period, during which participants were seated and completing the pre-simulation survey, was established as the baseline for EDA measurements (Horvers et al., 2021). Moreover, the EDA data were precisely synchronized with the video recordings of each participant using the built-in button on the E4 device. Participants were instructed to press this button before and after the tutorial simulation, and actual VS scenario simulation. This precaution ensured precise alignment of the two data channels, facilitating accurate syncing of EDA data with the qualitatively coded challenging/bothersome events ($N = 162$).

Once the EDA signals were acquired, the data were subjected to pre-processing using Ledalab (version 3.4.9), an open-source software platform implemented in MATLAB (Version R2023a) (Benedek & Kaernbach, 2010a, 2010b). The initial step in pre-processing involved a thorough visual inspection of the raw EDA signals. Subsequently, to mitigate high-frequency noise and movement artifacts, a Butterworth low-pass filter with a cut-off frequency of 1 Hz and an order of 1 was employed. Any remaining artifacts, such as those caused by movement or lost contact between the participant's skin and the electrodes or abnormally low values, were manually corrected through spline interpolation. Following pre-processing, a Continuous Decomposition Analysis (CDA) was performed to deconvolve the EDA signals into their tonic and phasic components. The phasic component, which serves as a sensitive indicator of stimulus-specific arousal (Chu et al., 2024)—such as challenging/bothersome events encountered within a virtual simulation—was subsequently extracted. The threshold for the amplitude of the phasic response was established at 0.05 μS, consistent with established precedents in the literature (Benedek & Kaernbach, 2010a, 2010b; Boucsein, 2012). All analyses of EDA focused exclusively on the continuous phasic component, thereby providing a continuous measure of the learners' experiences (Collins et al., 2019). To account for individual differences, these EDA measures were standardized (sEDA) using z-scores (Boucsein, 2012). The abbreviation sEDA will be utilized moving forward.

Distinct "before" and "after" periods were defined for each event to examine the changes in EDA and ascertain the physiological responses associated with challenging/bothersome events. The "before" period was crucial for capturing fluctuations in EDA that depict the participant's physiological state prior to the event. This period helped to identify any consistent arousal levels that might be present throughout the simulation, rather than those solely induced by the events. Conversely, the "after" period was examined to observe potential increases in arousal directly attributable to challenging/bothersome events. To accurately capture these periods, we established minimum and maximum duration thresholds for each "before" and "after" period: a minimum of 5 seconds to ensure the entire event was included (as events were instantaneous, 5 seconds would allow us to capture EDA changes due to the event), and no more than 60 seconds to align with the peak-per-minute principle prevalent in EDA research (Horvers et al., 2021; Malmberg et al., 2019). This approach was designed to provide a balanced view of the physiological

responses before and after each event, mitigating any distortion from overlapping events. Due to such overlaps, 40 events were excluded to maintain the integrity of the arousal data and respect the qualitative categorization of events, ultimately narrowing the dataset to 122 challenging/bothersome events from 20 participants. A schematic of these thresholds and the rules applied to the periods of interest for each event is depicted in Figure 2.

EDA latency was also considered to accommodate the inherent physiological delay following exposure to an arousing stimulus (Boucsein, 2012). Consequently, a delay of 3 seconds was applied to the onset of each designated before and after period surrounding the challenging/bothersome events to ensure an accurate capture of the EDA responses (see Figure 3 for a visual representation) (Boucsein, 2012; Caruelle et al., 2019; Sjouwerman & Lonsdorf, 2019). Following this adjustment, a weighted average was calculated for the before and after periods of the events. This method allowed us to normalize the data across varying lengths of these periods, adhering to the established minimum and maximum thresholds yet accommodating any duration within these limits.

Supplemental Material 3

Detailed Breakdown of Each Qualitative Observation and Their Frequencies Group by Participant Numbers

- See attached excel sheet titled "Supplemental Material 3"

Supplemental Material 4

Multiple Linear Regression with Cluster Robust Errors: Examining changes in sEDA across all event categories.

					95% CI	
**Predictor Variables**	***b***	**Robust *SE***	***t***	***p***	***LL***	***UL***
Baseline sEDA	-0.04	0.05	-0.76	0.45	-0.14	0.06
Intercept	0.03	0.09	0.37	0.72	-0.15	0.22
Software-related Restrictions & Bugs sEDA Change	Ref	Ref	Ref	Ref	Ref	Ref
Confusion/Lack of Success sEDA Change	0.12	0.11	1.14	0.27	-0.10	0.34
Negative Affect sEDA Change	0.13	0.11	1.120	0.28	-0.11	0.36
Technical Errors sEDA Change	-0.09	0.24	-0.38	0.71	-0.60	0.42
Neglect of Instruction sEDA Change	0.12	0.25	0.48	0.64	-0.40	0.63
Other sEDA Change	0.06	0.11	0.54	0.60	-0.17	0.29

Note. N = 67; R^2 = 0.04; Root-mean-square deviation = 0.41; *b = coefficient;* Robust *SE* = Robust standard error; CI = confident interval; *LL* = lower limit: *UL* = upper limit. Ref indicates the reference category to which comparisons were made. Software-related restrictions & bugs included flaws, errors, or faults in the operation of the VS that produced an incorrect or unexpected result; Confusion/lack of success was regarded as difficulty trying to understand what was going on and/or how to proceed within the VS; Negative affect included feeling visibly upset or irritated due to the inability to achieve a desired outcome; Technical errors included unintentional actions or errors that were not supposed to be conducted; Neglect of instruction were intentional actions that went against the instructions that were being provided; The Other category had general observations that do not fall under the above categories. All variations of the above comparison (using each category as a reference point) were run and yielded non-significant results.

Supplemental Material 5

Multiple Linear Regression with Cluster Robust Errors: Examining if change in a category's EDA from before and after challenging/bothersome events influences students' performance relative to other event categories

					95% CI	
**Performance Score**	***b***	**Robust *SE***	***t***	***p***	***LL***	***UL***
Change in Category EDA	11.96	13.62	0.88	0.39	-16.54	40.46
Software-related Restrictions & Bugs sEDA Change	Ref	Ref	Ref	Ref	Ref	Ref
Confusion/Lack of Success sEDA Change	-11.90	19.02	-0.63	0.54	-51.70	27.91
Negative Affect sEDA Change	62.87	65.04	0.97	0.35	-73.26	198.99
Technical Errors sEDA Change	-20.28	15.55	-1.30	0.21	-52.82	12.26
Neglect of Instruction sEDA Change	-1.57	15.12	-0.10	0.92	-33.22	30.07
Other sEDA Change	-13.70	13.41	-1.02	0.32	-41.76	14.37
Baseline sEDA	-2.41	4.35	-0.56	0.59	-11.51	6.68
Constant	62.38	5.84	10.68	0.00	50.16	74.61

					95% CI	
**Pairwise Comparisons**	***b***	**Robust *SE***	***t***	***p***	***LL***	***UL***
Confusion/Lack of Success sEDA Change vs Software-related Restrictions & Bugs sEDA Change	-11.90	19.02	-0.63	0.54	-51.70	27.91
Negative Affect sEDA Change vs Software-related Restrictions & Bugs sEDA Change	62.87	65.04	0.97	0.35	-73.26	198.99
Technical Errors sEDA Change vs Software-related Restrictions & Bugs sEDA Change	-20.28	15.55	-1.30	0.21	-52.82	12.26
Neglect of Instruction sEDA Change vs Software-related Restrictions & Bugs sEDA Change	-1.57	15.12	-0.10	0.92	-33.22	30.07
Other sEDA Change vs Software-related Restrictions & Bugs sEDA Change	-13.70	13.41	-1.02	0.32	-41.76	14.37
Negative Affect sEDA Change vs Confusion/Lack of Success sEDA Change	74.76	65.23	1.15	0.27	-61.77	211.30
Technical Errors sEDA Change vs Confusion/Lack of Success sEDA Change	-8.39	11.12	-0.75	0.46	-31.66	14.89
Neglect of Instruction sEDA Change vs Confusion/Lack of Success sEDA Change	10.32	12.26	0.84	0.41	-15.33	35.97
Other sEDA Change vs Confusion/Lack of Success sEDA Change	-1.80	17.10	-0.11	0.92	-37.58	33.98
Technical Errors sEDA Change vs Negative Affect sEDA Change	-83.15	64.48	-1.29	0.21	-218.11	51.81
Neglect of Instruction sEDA Change vs Negative Affect sEDA Change	-64.44	63.64	-1.01	0.32	-197.65	68.77
Other sEDA Change vs Negative Affect sEDA Change	-76.56	61.19	-1.25	0.23	-204.63	51.50
Neglect of Instruction sEDA Change vs Technical Errors sEDA Change	18.71	5.69	3.29	0.00	6.80	30.62
Other sEDA Change vs Technical Errors sEDA Change	6.59	9.71	0.68	0.51	-13.73	26.90

Other sEDA Change<br>vs<br>Neglect of Instruction sEDA Change	-12.12	10.27	-1.18	0.25	-33.63	9.38

Note. N = 67; R^2 = 0.106; Root-mean-square deviation = 15.277; *b = coefficient;* Robust *SE* = Robust standard error; CI = confident interval; *LL* = lower limit: *UL* = upper limit. Ref indicates the reference category to which comparisons were made. $F(12, 19) = 7.990$, $p < 0.001$.

Supplemental Material 6

Multiple Linear Regression with Cluster Robust Errors: Examining if change in a category's EDA from before and after challenging/bothersome events influences students' rating of useability relative to other event categories

					95% CI	
**Useability**	***b***	**Robust *SE***	***t***	***p***	***LL***	***UL***
Change in Category EDA	3.61	8.34	0.43	0.67	-13.84	21.07
Software-related Restrictions & Bugs sEDA Change	Ref	Ref	Ref	Ref	Ref	Ref
Confusion/Lack of Success sEDA Change	-5.29	14.38	-0.37	0.72	-35.37	24.80
Negative Affect sEDA Change	-124.60	58.69	-2.12	0.05	-247.45	-1.76
Technical Errors sEDA Change	-5.29	11.05	-0.48	0.64	-28.40	17.83
Neglect of Instruction sEDA Change	-2.96	10.32	-0.29	0.78	-24.55	18.63
Other sEDA Change	-42.67	12.59	-3.39	0.00	-69.02	-16.31
Baseline sEDA	2.74	4.91	0.56	0.58	-7.54	13.03
Constant	64.66	6.07	10.65	0.00	51.96	77.37

					95% CI	
**Pairwise Comparisons**	***b***	**Robust *SE***	***t***	***p***	***LL***	***UL***
Confusion/Lack of Success sEDA Change vs Software-related Restrictions & Bugs sEDA Change	-5.29	14.38	-0.37	0.72	-35.37	24.80
Negative Affect sEDA Change vs Software-related Restrictions & Bugs sEDA Change	-124.60	58.69	-2.12	0.05	-247.45	-1.76
Technical Errors sEDA Change vs Software-related Restrictions & Bugs sEDA Change	-5.29	11.05	-0.48	0.64	-28.40	17.83
Neglect of Instruction sEDA Change vs Software-related Restrictions & Bugs sEDA Change	-2.96	10.32	-0.29	0.78	-24.55	18.63
Other sEDA Change vs Software-related Restrictions & Bugs sEDA Change	-42.67	12.59	-3.39	0.00	-69.02	-16.31
Negative Affect sEDA Change vs Confusion/Lack of Success sEDA Change	-119.32	56.09	-2.13	0.05	-236.72	-1.92
Technical Errors sEDA Change vs Confusion/Lack of Success sEDA Change	-0.00	11.55	-0.00	1.00	-24.17	24.16
Neglect of Instruction sEDA Change vs Confusion/Lack of Success sEDA Change	2.32	15.65	0.15	0.88	-30.44	35.09
Other sEDA Change vs Confusion/Lack of Success sEDA Change	-37.38	14.15	-2.64	0.02	-67.00	-7.76
Technical Errors sEDA Change vs Negative Affect sEDA Change	119.32	54.10	2.21	0.04	6.09	232.55
Neglect of Instruction sEDA Change vs Negative Affect sEDA Change	121.64	58.58	2.08	0.05	-0.96	244.25
Other sEDA Change vs Negative Affect sEDA Change	81.94	54.70	1.50	0.15	-32.55	196.43
Neglect of Instruction sEDA Change vs Technical Errors sEDA Change	2.33	9.84	0.24	0.82	-18.27	22.92
Other sEDA Change vs Technical Errors sEDA Change	-37.38	11.15	-3.35	0.00	-60.70	-14.05

Note. N = 67; R^2 = 0.17; Root-mean-square deviation = 13.87; *b = coefficient;* Robust *SE* = Robust standard error; CI = confident interval; *LL* = lower limit: *UL* = upper limit. Ref indicates the reference category to which comparisons were made. $F(12, 19) = 2.40$, $p = 0.04$. Note that the comparison between Neglect of Instruction sEDA Change and Negative Affect sEDA Change is **not** significant (p=0.052)

Supplemental Material 7

Multiple Linear Regression with Cluster Robust Errors: Examining if change in a category's EDA from before and after challenging/bothersome events influences students' rating of extrinsic cognitive load relative to other event categories

					95% CI	
**Extrinsic Cognitive Load**	***b***	**Robust *SE***	***t***	***p***	***LL***	***UL***
Change in Category EDA	1.13	0.45	2.52	0.02	0.19	2.08
Software-related Restrictions & Bugs sEDA Change	Ref	Ref	Ref	Ref	Ref	Ref
Confusion/Lack of Success sEDA Change	-0.07	0.95	-0.07	0.95	-2.05	1.92
Negative Affect sEDA Change	4.14	3.12	1.33	0.20	-2.39	10.67
Technical Errors sEDA Change	-1.22	0.42	-2.92	0.01	-2.09	-0.34
Neglect of Instruction sEDA Change	-2.62	0.65	-4.05	0.00	-3.98	-1.27
Other sEDA Change	1.31	1.46	0.89	0.38	-1.75	4.37
Baseline sEDA	0.05	0.35	0.14	0.89	-0.68	0.77
Constant	2.08	0.29	7.06	0.00	1.46	2.70

					95% CI	
**Pairwise Comparisons**	***b***	**Robust *SE***	***t***	***p***	***LL***	***UL***
Confusion/Lack of Success sEDA Change vs Software-related Restrictions & Bugs sEDA Change	-0.07	0.95	-0.07	0.95	-2.05	1.92
Negative Affect sEDA Change vs Software-related Restrictions & Bugs sEDA Change	4.14	3.12	1.33	0.20	-2.39	10.67
Technical Errors sEDA Change vs Software-related Restrictions & Bugs sEDA Change	-1.22	0.42	-2.92	0.01	-2.09	-0.34
Neglect of Instruction sEDA Change vs Software-related Restrictions & Bugs sEDA Change	-2.62	0.65	-4.05	0.00	-3.98	-1.27
Other sEDA Change vs Software-related Restrictions & Bugs sEDA Change	1.31	1.46	0.89	0.38	-1.75	4.37
Negative Affect sEDA Change vs Confusion/Lack of Success sEDA Change	4.20	3.29	1.28	0.22	-2.68	11.09
Technical Errors sEDA Change vs Confusion/Lack of Success sEDA Change	-1.15	0.96	-1.20	0.24	-3.15	0.85
Neglect of Instruction sEDA Change vs Confusion/Lack of Success sEDA Change	-2.56	1.21	-2.11	0.05	-5.09	-0.02
Other sEDA Change vs Confusion/Lack of Success sEDA Change	1.37	1.93	0.71	0.49	-2.67	5.42
Technical Errors sEDA Change vs Negative Affect sEDA Change	-5.35	2.97	-1.80	0.09	-11.57	0.86
Neglect of Instruction sEDA Change vs Negative Affect sEDA Change	-6.76	3.20	-2.11	0.05	-13.46	-0.06
Other sEDA Change vs Negative Affect sEDA Change	-2.83	3.35	-0.85	0.41	-9.84	4.18
Neglect of Instruction sEDA Change vs Technical Errors sEDA Change	-1.41	0.46	-3.05	0.01	-2.37	-0.44
Other sEDA Change vs Technical Errors sEDA Change	2.52	1.59	1.59	0.13	-0.80	5.84

Other sEDA Change<br>vs<br>Neglect of Instruction sEDA Change	3.93	1.76	2.24	0.04	0.25	7.60

Note. N = 67; $R^2 = 0.09$; Root-mean-square deviation = 1.39; *b = coefficient;* Robust *SE* = Robust standard error; CI = confident interval; *LL* = lower limit: *UL* = upper limit. Ref indicates the reference category to which comparisons were made. $F(12, 19) = 3.71$, $p = 0.01$.

References

Ahmed, S. K. (2024). The pillars of trustworthiness in qualitative research. *Journal of Medicine, Surgery, and Public Health*, *2*, 100051. https://doi.org/10.1016/j.glmedi.2024.100051

Benedek, M., & Kaernbach, C. (2010a). A continuous measure of phasic electrodermal activity. *Journal of Neuroscience Methods*, *190*(1), 80–91. https://doi.org/10.1016/j.jneumeth.2010.04.028

Benedek, M., & Kaernbach, C. (2010b). Decomposition of skin conductance data by means of nonnegative deconvolution. *Psychophysiology*, *47*(4), 647–658. https://doi.org/10.1111/j.1469-8986.2009.00972.x

Borrego, A., Latorre, J., Alcañiz, M., & Llorens, R. (2019). Reliability of the empatica e4 wristband to measure electrodermal activity to emotional stimuli. *2019 International Conference on Virtual Rehabilitation (ICVR)*, 1–2. https://doi.org/10.1109/ICVR46560.2019.8994546

Boucsein, W. (2012). *Electrodermal Activity*. Springer Science & Business Media.

Caruelle, D., Gustafsson, A., Shams, P., & Lervik-Olsen, L. (2019). The use of electrodermal activity (EDA) measurement to understand consumer emotions – A literature review and a call for action. *Journal of Business Research*, *104*, 146–160. https://doi.org/10.1016/j.jbusres.2019.06.041

Chu, B., Marwaha, K., Sanvictores, T., Awosika, A. O., & Ayers, D. (2024). Physiology, Stress Reaction. In *StatPearls*. StatPearls Publishing. http://www.ncbi.nlm.nih.gov/books/NBK541120/

Collins, J., Regenbrecht, H., Langlotz, T., Said Can, Y., Ersoy, C., & Butson, R. (2019). Measuring cognitive load and insight: A methodology exemplified in a virtual reality

learning context. *2019 IEEE International Symposium on Mixed and Augmented Reality (ISMAR)*, 351–362. https://doi.org/10.1109/ISMAR.2019.00033

Empatica Inc. (2021, October 21). *Empatica | User Manuals*. User Manuals. https://www.empatica.com/manuals

Garbarino, M., Lai, M., Bender, D., Picard, R. W., & Tognetti, S. (2014). Empatica E3—A wearable wireless multi-sensor device for real-time computerized biofeedback and data acquisition. *2014 4th International Conference on Wireless Mobile Communication and Healthcare - Transforming Healthcare Through Innovations in Mobile and Wireless Technologies (MOBIHEALTH)*, 39–42. https://doi.org/10.1109/MOBIHEALTH.2014.7015904

Horvers, A., Tombeng, N., Bosse, T., Lazonder, A. W., & Molenaar, I. (2021). Detecting Emotions through Electrodermal Activity in Learning Contexts: A Systematic Review. *Sensors*, *21*(23), Article 23. https://doi.org/10.3390/s21237869

Lincoln, Y. S., & Guba, E. G. (1985). *Naturalistic inquiry*. SAGE.

Malmberg, J., Järvelä, S., Holappa, J., Haataja, E., Huang, X., & Siipo, A. (2019). Going beyond what is visible: What multichannel data can reveal about interaction in the context of collaborative learning? *Computers in Human Behavior*, *96*, 235–245. https://doi.org/10.1016/j.chb.2018.06.030

Schuurmans, A. A. T., de Looff, P., Nijhof, K. S., Rosada, C., Scholte, R. H. J., Popma, A., & Otten, R. (2020). Validity of the Empatica E4 Wristband to Measure Heart Rate Variability (HRV) Parameters: A Comparison to Electrocardiography (ECG). *Journal of Medical Systems*, *44*(11), 190. https://doi.org/10.1007/s10916-020-01648-w

Sjouwerman, R., & Lonsdorf, T. B. (2019). Latency of skin conductance responses across stimulus modalities. *Psychophysiology*, *56*(4), e13307. https://doi.org/10.1111/psyp.13307
